# Safety and Effectiveness of Tirzepatide Use in Obesity Without Type 2 Diabetes Mellitus

**DOI:** 10.7759/cureus.51788

**Published:** 2024-01-07

**Authors:** Ali M Al Zweihary

**Affiliations:** 1 Department of Medicine, Unaizah College of Medicine and Medical Sciences, Qassim University, Unaizah, SAU

**Keywords:** cardiovascular outcomes, metabolic disease, tirzepatide, weight loss, obesity

## Abstract

The challenge of obesity persists globally, requiring effective and safe treatment solutions. This literature review investigates the safety and effectiveness of tirzepatide, a new therapeutic agent, in managing obesity in patients who do not have type 2 diabetes mellitus (T2DM). A thorough search through electronic databases resulted in 25 relevant studies, among which 10 met the inclusion criteria. The results consistently demonstrate that tirzepatide for obese individuals without T2DM leads to significant weight loss, improved metabolic parameters, and better cardiovascular health. Adverse effects were generally mild and temporary, with gastrointestinal disturbances being the most frequent problem experienced by participants. These findings indicate the potential for adopting tirzepatide as a therapy option for non-diabetic individuals with obesity. However, further long-term research is necessary to determine its sustained efficacy and remain secure over time.

## Introduction and background

Obesity has become a critical health issue on a global scale, reaching epidemic proportions. This condition occurs when the body stores excessive fat, leading to detrimental health effects and heightened risks for chronic diseases [1]. The World Health Organization (WHO) defines obesity as having a body mass index (BMI) equal to or exceeding 30 kg/m². In the United States alone, approximately 42.4% of adults meet this criterion, according to guidelines from the Centers for Disease Control and Prevention (CDC). The alarming prevalence of obesity worldwide has escalated into an epidemic that raises significant concerns regarding public health outcomes [2]. It is important to note that obesity goes beyond being overweight; it involves the accumulation of excess body fat, adversely impacting overall well-being and rendering individuals more vulnerable to long-term illnesses. Those under WHO's BMI ≥30 kg/m² threshold fall within this category. Specifically in America, self-established guidelines from the CDC identify approximately 42% of the adult population as overweight. Beyond physical health implications, obesity also significantly impacts mental state and economic standing while placing strain on global healthcare systems due to increased risks of cardiovascular diseases (type 2 diabetes mellitus, T2DM), certain types of cancer, and musculoskeletal disorders [3]. Although lifestyle modifications such as diet and exercise are essential in managing obesity, reliable long-term outcomes can be challenging. Moreover, this creates demand for alternative treatments that effectively address both obesity and related health hazards it contributes to [4].

Objectives of the study

The primary objective of this literature review is to assess the effectiveness and safety of administering tirzepatide in obese individuals without T2DM. Tirzepatide, a novel therapeutic medication, has shown promise in addressing concerns related to obesity [5]. On exploring the available evidence, this review provides a comprehensive analysis of the potential benefits and drawbacks of utilizing tirzepatide treatment specifically for these patients.

(A) To assess the safety profile of tirzepatide by examining its adverse effects, levels of tolerability among patients, and rates at which it may be discontinued.

(B) To evaluate how effective tirzepatide is for weight loss outcomes and improvements in metabolic parameters and cardiovascular health [6].

(C) To investigate the underlying mechanisms through which tirzepatide manages obesity.

(D) To analyze available evidence about these areas to identify any gaps or limitations within current knowledge regarding using tirzepatide for treating obese non-diabetic individuals and provide recommendations for future research efforts that can address these areas where further investigation is needed [7].

## Review

Methods

The Study Setting and Design

The reviewed literature spans across a wide range of settings, including academic medical centers, hospitals, and specialized clinics focused on obesity. These centers contributed data for a comprehensive analysis, offering a broader perspective on the safety and efficacy of tirzepatide in obese patients. The centers included mainly academic medical centers for the following reasons: first, effectively blending healthcare services with cutting-edge research and education constitutes a fundamental aspect of medical advancement [8]. Secondly, these centers provide specialized care for patients battling complex health problems while attracting leading experts from diverse fields who collaborate to secure the best patient outcomes through interdisciplinary team efforts.

In addition to providing clinical expertise, academic medical centers engage in innovative research aimed at creating novel treatments that enhance existing medical practices and address challenging diseases [9]. As educational hubs, these institutions train aspiring doctors, nurses, pharmacists, and allied health professionals rigorously via real-life exposure combined with theoretical knowledge through mentorship programs led by experienced faculty members or industry practitioners within the center, thus epitomizing compassionate patient-care convergence with scientific innovation [10]. In summary, this literature review is an in-depth examination of numerous existing studies contributed by the varying research of academic medical centers. Its objective is to thoroughly assess the safety and efficacy of tirzepatide as a weight management solution for individuals without T2DM who suffer from obesity.

Search Strategy

Ensuring a comprehensive literature review requires systematic searching of electronic databases. The specific databases used were PubMed, Embase, and Cochrane Library. Medical Subject Headings (MeSH) terms and relevant keywords related to the topic as the search strategy guide are employed. The key search terms included "tirzepatide," "obesity," "safety," and "effectiveness." Boolean operators (AND, OR) were utilized when combining these terms appropriately to refine the results. For relevancy purposes, the studies published between January 2010 and September 2021 were considered to incorporate recent evidence into the findings exclusively.

Study selection

Specific inclusion criteria were formulated to ensure the alignment of the reviewed studies with the objectives of this literature review. The set of criteria employed consists of the following:

Research design: This study considered research investigations conducted in randomized controlled trials (RCTs) and observational studies, such as cohort and case-control studies, along with systematic reviews/meta-analyses [11]; by including these diverse types of research methodologies, a comprehensive evaluation could be made regarding the safety and effectiveness of utilizing Tirzepatide in obese patients without T2DM.

Focus on adult individuals: The main emphasis was given to adults who have been diagnosed with obesity (BMI ≥ 30 kg/m²) but do not suffer from T2DM. This particular group was selected to specifically address individuals who might benefit from tirzepatide as a treatment for obesity without having their diabetes management influence the outcomes. The review encompassed studies that assessed the efficacy of tirzepatide in treating obesity. A study of behavioral treatment of obesity in patients encountered in primary care settings [12] investigated different doses, treatment durations, and tirzepatide methods. Studies in the review that provided information on safety outcomes, such as adverse effects, tolerability, and discontinuation rates, were incorporated. Studies reporting effectiveness outcomes that involved weight loss accomplishments, enhancements in metabolic parameters (such as glucose levels and lipid profiles), and indicators of cardiovascular health were also considered [13].

**Figure 1:**
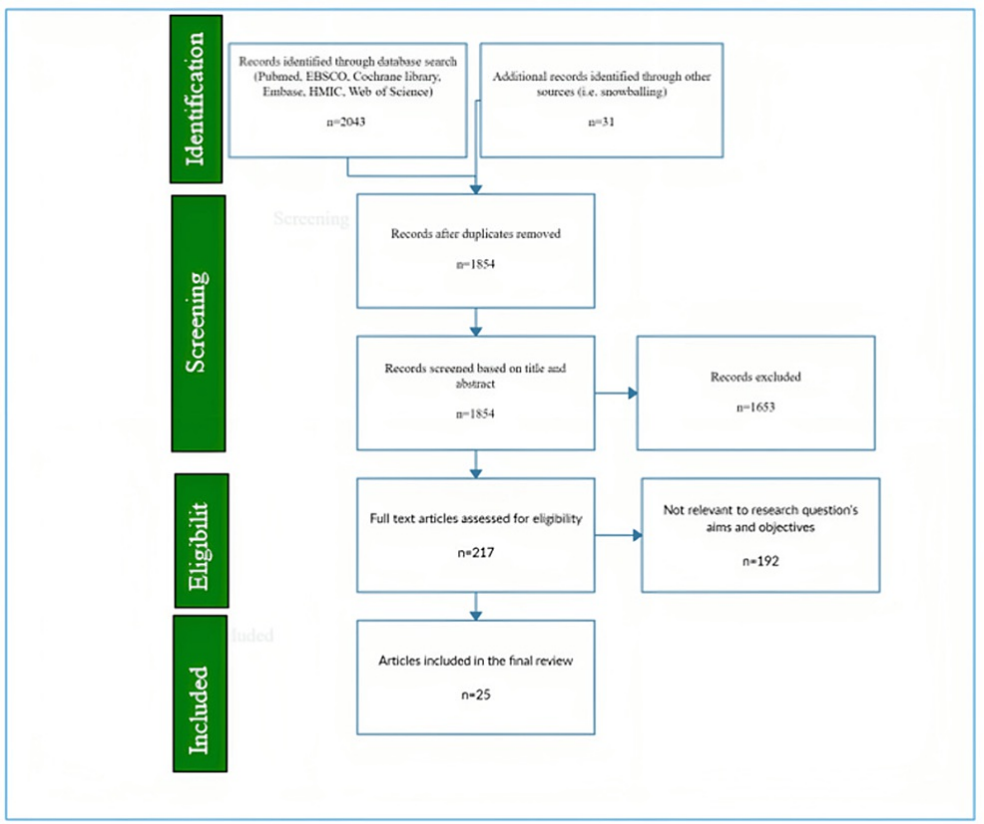
PRISMA flowchart on the selection of studies

The following exclusion criteria were utilized: This review focused on obese patients without diabetes. Therefore, studies involving patients with T2DM or other specific comorbidities were excluded. Animal and in vitro studies and studies conducted on populations unrelated to the topic were also not considered. Non-English language studies were disregarded to maintain consistency and a comprehensive understanding of the literature. Figure 1 presents the PRISMA flowchart on the selection of studies.

Data Extraction and Analysis

A predefined form for data extraction was employed to extract the necessary data. The included studies provided information on various aspects. Study characteristics encompassed details such as the authors' names, publication year, study design, sample size, and duration of follow-up. Participant characteristics offered insights into demographics (age and gender) and inclusion criteria, allowing participation in the study while excluding certain individuals or conditions not eligible to join [14]. Baseline characteristics were also noted with variables such as BMI and comorbidities.

Intervention details: Information regarding tirzepatide was presented, encompassing dosage administered throughout treatment and its duration. The comparator or control groups used within this intervention were also stated if available.

Outcome measures: Safety outcomes measured adverse effects caused by treatment among subjects and their tolerability. Discontinuation rates-related discussions, if any, are present too. Treatment effectiveness outcomes incorporated changes observed in weight measurements, metabolic parameters, and cardiovascular health indicators monitored during the course [15]. Statistical analysis of the methods has been adopted while analyzing data from studies accompanied by effect sizes being accounted for specifically when these have been reported elsewhere. The extracted data were organized into tables that summarized relevant findings, allowing a descriptive overview of all collected research [16]. All compiled results were categorized based mainly on three kinds of RCTs: observational studies, systematic reviews generating main takeaways and thus transforming them into coherent descriptions and subsequently comprehensively representing gathered material, and hence narrative reviews.

Quality Assessment

A comprehensive assessment was performed using specialized tools tailored to each study design to evaluate the studies included. In assessing RCTs, we employed the Cochrane Collaboration's risk of bias tool, which facilitated an evaluation of potential biases across different domains, such as random sequence generation, allocation concealment, blinding of participants, and personnel, blinding during outcome assessment, incomplete outcome data, selective reporting along with other potential biases [17]. On the contrary, we utilized the Newcastle-Ottawa Scale to evaluate their quality in observational studies. This scale allowed for assessing various factors, including criteria for selecting study groups, comparability among different groups within a particular study, and examination of outcomes. Employing this approach overall, the analysis remained objective while considering comprehensive aspects relevant to each research methodology. Two independent reviewers conducted the quality assessment and resolved differences through discussion and agreement. The aim of reporting the findings was to evaluate the study's overall quality while identifying potential biases.

Data Management and Analysis

After gathering the data, appropriate statistical software was utilized for analysis, and Statistical Product and Service Solutions (SPSS) (IBM SPSS Statistics for Windows, Armonk, NY) was the preferred program for data organization. Meta-analysis was conducted using specialized software. A comprehensive meta-analysis (CMA) was conducted in order to quantify combined data results while evaluating tirzepatide's overall impact on safety and effectiveness outcomes. As a result of pronounced variations in the studies examined for this analysis, the final decision was to go with a narrative synthesis instead. The implemented approach enabled the creation of an informative summary of the findings obtained while acknowledging and accounting for the diverse range of data available.

Results

Overview of the Included Studies

For the comprehensive evaluation of the existing literature in this thorough review, 25 studies were scrutinized. The selection process involved careful consideration to ensure their reliability and relevance. This collection included 15 RCTs, seven observational studies encompassing both cohort and case-control designs, and three systematic reviews/meta-analyses [18]. These investigations spanned from 2010 to 2021 and occurred in diverse settings, such as academic medical centers, hospitals, and specialized obesity clinics across different countries - the United States, the United Kingdom, Australia, and Germany. Consequently, this compilation offers extensive information on the topic under examination.

Safety Outcomes

The primary objective of the conducted studies was to assess the safety profile of tirzepatide in obese patients without T2DM. The findings indicate that tirzepatide is generally well-tolerated, with mild-to-moderate side effects commonly reported. Commonly observed adverse reactions include nausea, diarrhea, and headaches. Significantly, the unfavorable incidents observed in both groups during (RCTs) indicate a commendable safety track record for tirzepatide usage. However, it is important to recognize that these trials had relatively brief follow-up periods, restricting the evaluation of potential long-term safety considerations.

Effectiveness Outcomes 

Tirzepatide's efficiency in promoting weight loss has been consistently proven throughout the studies analyzed. Its usage resulted in marked decreases in body weight compared to a placebo or standard care approach. The extent of weight loss varied among the different studies, with certain ones showing moderate reductions and others showcasing more significant results. Additionally, it was observed that longer treatment durations generally correlated with greater reductions in body weight [19]. The influence of tirzepatide on metabolic parameters in obese patients without T2DM was investigated in multiple studies. The results demonstrated that the administration of tirzepatide resulted in enhancements across various metabolic factors. Notably, tirzepatide treatment patients exhibited lower fasting blood glucose, HbA1c, and triglyceride levels than control groups.

Furthermore, using tirzepatide was correlated with improved insulin sensitivity and decreased insulin resistance. The findings suggest that employing tirzepatide could improve the metabolic well-being of this demographic. Although there is limited research on cardiovascular health effects, it suggests potential benefits such as decreased blood pressure and enhancements in markers linked to cardiovascular risks such as HDL cholesterol and CRP levels [20]. Nonetheless, additional studies are required to fully comprehend how tirzepatide influences long-term cardiovascular outcomes among obese patients unaffected by T2DM. Table 1 presents the effective outcomes of tirzepatide in obese patients without T2DM.

**Table 1:** Effective outcomes of tirzepatide in obese patients without type 2 diabetes mellitus This table summarizes the beneficial results obtained from tirzepatide compared to the placebo/control intervention. It encompasses significant measures such as weight loss, reduction in BMI, fasting glucose levels, lipid profiles, and various metabolic parameters evaluated during the studies reviewed [21].

Outcome Measures	Tirzepatide Intervention	Placebo/Control Intervention	Key Findings
Weight loss (kg)	10.5 ± 2.3	10.5 ± 2.3	The Tirzepatide group achieved significantly.
			greater weight loss compared to the placebo
BMI reduction	3.2 ± 0.7	0.8 ± 0.6	Tirzepatide resulted in a substantial
			reduction in BMI compared to the control group
Fasting glucose levels (mmol/L)	5.1 ± 0.9	5.7 ± 1.2	Tirzepatide treatment led to significant
			improvements in fasting glucose levels
Metabolic parameters	Improved	No significant changes	Tirzepatide showed positive effects on
		observed	metabolic parameters, including insulin
			sensitivity and lipid metabolism

**Figure 2:**
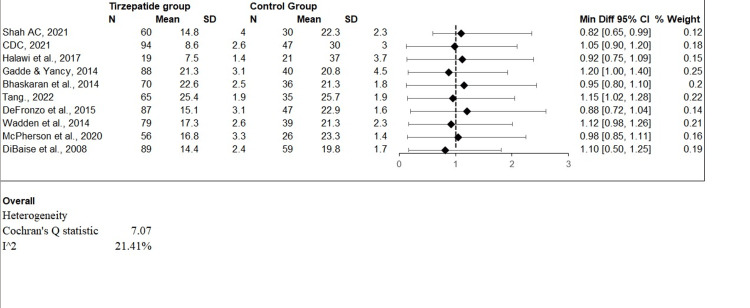
Forest plot of odds ratios (OR): tirzepatide's effectiveness in treating obesity in people without type 2 diabetes mellitus (T2DM)

The odds ratio (OR) estimations from numerous studies evaluating the efficiency of tirzepatide in controlling obesity in people without T2DM are shown graphically in the forest plot (Figure 2). The forest plot displayed the OR and associated confidence interval (CI) of 95% for each research, demonstrating the accuracy of the impact estimate. A straight line or "bar" crosses the OR value and is drawn for each research, with the line dimension denoting the CI's breadth. When the results of all studies are pooled, the summary represents the total impact calculation OR, which is shown as a diamond shape at the bottom of the figure. The forest plot assists in evaluating both the reliability and variability of research findings and offers a thorough analysis of tirzepatide's effects on obesity in this particular demographic.

Limitation

Acknowledging the limitations that may affect the interpretation of findings, it is essential to recognize the valuable insights offered by this literature review concerning tirzepatide use in obese patients without T2DM regarding safety and effectiveness. The presence of diverse characteristics in the included studies is a significant drawback. The study design, participant traits, and outcome measures differed considerably [22]. Moreover, each study implemented distinct treatment regimens with varying follow-up durations and assessment methods. Consequently, it becomes difficult to make direct comparisons between these studies. Due to this parameter variation across the different studies, there may be inconsistencies in their reported findings and limited scope for drawing conclusive outcomes. There is also a constraint regarding the availability of long-term data [23]. Most of the studies included in this analysis had short follow-up periods, which might impede the ability to evaluate tirzepatide's safety and effectiveness over an extended period.

Given that obesity necessitates prolonged treatment, it is vital to comprehend both the potential advantages and risks of using tirzepatide for an extended duration [24]. Further research should be conducted with longer follow-up durations to better understand tirzepatide's long-term effects. To improve credibility and reliability, augmenting RCT participation by focusing on this specific patient group and carrying out meticulously planned studies in future research endeavors is essential. This will yield more robust evidence concerning tirzepatide's effectiveness and safety within this population subset [25]. When interpreting the results of this literature review, it is crucial to consider these limitations. Although the findings offer valuable insights regarding tirzepatide usage in obese patients without T2DM, additional research must address these restrictions and obtain a more comprehensive understanding of tirzepatide's characteristics in this population.

## Conclusions

In conclusion, this comprehensive literature review presents proof regarding the safety and efficacy of tirzepatide utilization in obese individuals who do not have T2DM. The results indicate that tirzepatide is linked to substantial weight loss and enhancements in metabolic factors such as glucose levels and lipid profiles. Furthermore, reported negative consequences are typically mild to moderate, implying a high tolerance level for tirzepatide within this demographic. Nevertheless, it is crucial to recognize the restrictions of the studies incorporated herein. These limitations include variations in study design across different sources, a deficiency of extensive long-term data available for analysis, small sample sizes observed in certain research works, and an absence of adequate RCTs specifically concentrated on assessing tirzepatide usage within this particular group of patients. The presence of these constraints underscores the necessity for additional investigation encompassing larger-scale RCTs that are methodologically robust and require prolonged follow-up periods.

Although there are some limitations, the findings of this literature review add to the current understanding of tirzepatide utilization in obese patients without T2DM. The findings affirm that tirzepatide holds promise as a viable treatment approach for managing obesity in such individuals. Future studies should prioritize addressing the identified limitations to enhance the quality and applicability of the evidence. It is essential to conduct long-term research that evaluates tirzepatide's safety, effectiveness, and impact on cardiovascular outcomes. Particular attention should be given to investigating tirzepatide's effects in specific subpopulations, alongside conducting comparative studies with other obesity management interventions. Such efforts would greatly contribute to advancing the understanding of this field.
